# SIRT1 mediated gastric cancer progression under glucose deprivation through the FoxO1-Rab7-autophagy axis

**DOI:** 10.3389/fonc.2023.1175151

**Published:** 2023-05-24

**Authors:** Mengke Zhu, Chao Wei, Haijiang Wang, Shangning Han, Lindi Cai, Xiaowen Li, Xinhua Liao, Xiangming Che, Xuqi Li, Lin Fan, Guanglin Qiu

**Affiliations:** ^1^Department of General Surgery, The First Affiliated Hospital of Xi’an Jiaotong University, Xi’an, Shaanxi, China; ^2^Clinical Medicine Teaching and Research Section, Xi’an Health School, Xi’an, Shaanxi, China; ^3^Department of General Surgery, The Second Affiliated Hospital of Xi’an Jiaotong University, Xi’an, Shaanxi, China

**Keywords:** gastric cancer, SIRT1-FoxO1-Rab7, autophagy, target treatment, glucose deprivation

## Abstract

**Purpose:**

Silent mating type information regulator 2 homolog 1 (SIRT1) and autophagy have a two-way action (promoting cell death or survival) on the progression and treatment of gastric cancer (GC) under different conditions or environments. This study aimed to investigate the effects and underlying mechanism of SIRT1 on autophagy and the malignant biological behavior of GC cells under conditions of glucose deprivation (GD).

**Materials and methods:**

Human immortalized gastric mucosal cell GES-1 and GC cell lines SGC-7901, BGC-823, MKN-45 and MKN-28 were utilized. A sugar-free or low-sugar (glucose concentration, 2.5 mmol/L) DMEM medium was used to simulate GD. Additionally, CCK8, colony formation, scratches, transwell, siRNA interference, mRFP-GFP-LC3 adenovirus infection, flow cytometry and western blot assays were performed to investigate the role of SIRT1 in autophagy and malignant biological behaviors (proliferation, migration, invasion, apoptosis and cell cycle) of GC under GD and the underlying mechanism.

**Results:**

SGC-7901 cells had the longest tolerance time to GD culture conditions, which had the highest expression of SIRT1 protein and the level of basal autophagy. With the extension of GD time, the autophagy activity in SGC-7901 cells also increased. Under GD conditions, we found a close relationship between SIRT1, FoxO1 and Rab7 in SGC-7901 cells. SIRT1 regulated the activity of FoxO1 and upregulated the expression of Rab7 through deacetylation, which ultimately affected autophagy in GC cells. In addition, changing the expression of FoxO1 provided feedback on the expression of SIRT1 in the cell. Reducing SIRT1, FoxO1 or Rab7 expression significantly inhibited the autophagy levels of GC cells under GD conditions, decreased the tolerance of GC cells to GD, enhanced the inhibition of GD in GC cell proliferation, migration and invasion and increased apoptosis induced by GD.

**Conclusion:**

The SIRT1-FoxO1-Rab7 pathway is crucial for the autophagy and malignant biological behaviors of GC cells under GD conditions, which could be a new target for the treatment of GC.

## 1 Introduction

Gastric cancer (GC) is a commonly diagnosed malignancy ranked among the top five in morbidity and mortality of all cancers worldwide (1). Since the prognosis of GC patients with advanced or metastatic diseases remains poor, clarifying the potential molecular mechanism of its occurrence and development and finding new therapeutic targets are of great significance for improving patient outcomes.

Silent mating type information regulator 2 homolog 1 (SIRT1) and autophagy have a two-way action (promoting cell death or survival) on the development and treatment of GC under different conditions. SIRT1 is a class-III histone deacetylase (HDAC), a Nicotinamide adenine dinucleotide+ (NAD+)-dependent enzyme and a prognostic factor for many tumors, including GC (2, 3). It was reported to be vital for GC proliferation, migration, invasion, epithelial-mesenchymal transition (EMT), apoptosis and resistance to chemotherapy (4–6). Thus, SIRT1 is considered an important therapeutic target. However, its effect on GC cell proliferation, migration, invasion, and apoptosis under sugar deprivation condition is still unclear. Our previous research showed that GC tissues exhibit higher levels of autophagic activity and expression of Beclin-1 and SIRT1 than normal adjacent gastric mucosa (7). In addition, SIRT1 expression was positively correlated with Beclin-1 expression, and both SIRT1 and Beclin-1 were significant factors that affected the prognosis of GC patients. Thus, we believe that autophagy and SIRT1 play an essential role in the occurrence and development of GC. Previous researches showed that SIRT1 participated in the regulation of autophagy by modifying ATG proteins (8), *via* the SIRT1-FoxO1-Rab7 axis (Sirt1-mediated deacetylation of FoxO1 and upregulation of Rab7) (9) and modifying other molecules such as histone H4 (at lysine residue 16;H4K16ac) (10), Forkhead box O1(FoxO1) (11), Forkhead box O3(FoxO3) (12), E2F transcription factor 1(E2F1) (13), p73 (14), Peroxisome proliferator-activated receptor-γ coactivator-1α(PGC1α) (15), S6 kinase (S6K) (16), Nuclear factor-k-gene binding (NF-κB) (17), p53 (18) and tuberous sclerosis complex 2 (TSC2) (19). However, the pathway *via* which SIRT1 regulates autophagic activities in GC cells and its effects on the malignant biological behavior of GC cells under glucose deprivation (GD) conditions remain unclear.

Therefore, this present study aimed to clarify the effects and mechanism of SIRT1 and the SIRT1-FoxO1-Rab7 axis on the autophagy activity and cell biological behavior of GC cells under GD conditions.

## 2 Materials and methods

### 2.1 Reagents

Sugar-free DMEM medium (Glucose concentration 0mmol/L, GIBCO, USA), DMEM medium (glucose concentration 25mmol/L, HyClone USA), annexin V-FITC Apoptosis Detection Kit (Beijing 4A Biotech Co., Ltd), CCK8 Kit (Wuhan Boster Biological Technology Co., Ltd), siRNA Lyophilized Powder (Shanghai GenePharma Co., Ltd), mRFP-GFP-LC3 adenovirus (Han Heng Biotechnology Co., Ltd), rabbit anti-human β-actin, LC3AB, Cleaved Caspase 3, FoxO1 and Rab7 monoclonal antibody (Cell Signaling Technology, USA), Beclin-1 Rabbit anti-human monoclonal antibody, P62 mouse anti-human monoclonal antibody, Caspase 3 rabbit anti-human monoclonal antibody (Abcam, USA), rabbit anti-human SIRT1, Ac-FoxO1 polyclonal antibody (Santa Cruz, USA), horseradish peroxidase-labeled goat anti-mouse secondary antibodies, goat anti-rabbit secondary antibodies(Pierce, USA).

### 2.2 Cell lines

Human immortalized gastric mucosal epithelial cell GES-1 and GC cell lines SGC-7901, BGC-823, MKN-45and MKN-28 obtained from the Laboratory Animal Center of the Fourth Military Medical University were used in this study. These five cell lines were cultured in DMEM medium containing 10% fetal calf serum at 37 °C, 5% carbon dioxide concentration and appropriate humidity.

### 2.3 Cell viability analysis

We used the CCK8 kit test for cell viability assay. After transfection of siRNA, each group of cells was cultured for 24 hours in a 96-well plate with 5000 cells/well and replaced with sugar or sugar-free DMEM medium for 0, 24, 48, 72 and 96 hours. Then, each well was changed to fresh medium with 10 μl CCK-8 solution and cultured for about 2 hours. A negative control containing medium and CCK-8 but no cells in the wells was also set up. The OD value of each well at a wavelength of 450 nm was detected and corrected with the OD value of the negative control group.

### 2.4 Plate clone formation

After transfection with siRNA, 200 cells/well were seeded in a 6-well plate containing 5 ml of 10% FBS in DMEM or sugar-free DMEM medium and cultured for about 14-21 days. After the medium was discarded, the cells were washed twice with PBS, fixed with methanol for 15 minutes, and stained with Giemsa for 15-30 minutes. An effective clone was defined as >50 cells. The clone formation rate was calculated using the following equation. The experiment was repeated 3 times.


clone formation rate=(number of clones/number of inoculated cells)× 100%

### 2.5 Scratches

After transfection with siRNA, 5×10^5^ cells were seeded in each well of a 6-well plate. The cells were fused to about 90% and scored with a 100 μl gun tip perpendicular to the bottom of the six-well plate. After scratching, the cells were washed with PBS to remove the detached cells. They were then observed under a microscope and photographed (0 hours) to record the distance between the two sides of the wound at 24, 48 and 72 hours, respectively. The images taken were used to record the distance between both sides of the scar, and a software was used to analyze the cell migration distance of each group of cells at 0, 24, 48, and 72 hours. The relative migration distance was calculated using the following formula.


migration distance=(scratch width at 0,24,48,and 72 hours−scratch width)/2

### 2.6 Transwell migration and invasion assays

Transwell migration and invasion experiments were performed with Transwell chambers (Millipore, 8μm/well). The microporous membrane of the upper chamber was covered with or without Matrigel (Becton Dickinson Labware) for the invasion and migration experiments, respectively. The cells of each group transfected with siRNA were then collected, to which 500μl of DMEM or low-glucose DMEM medium containing 20% FBS was added to the 24-well plate (lower chamber). Next, 6×10^4^ or 1×10^5^ cells were seeded in serum-free DMEM or low-glucose DMEM medium in the chamber for migration and invasion experiments, respectively. After culturing for 12 hours (migration assay) and 36 hours (invasion assay), the cells in the upper layer of the microporous membrane were removed using cotton swabs. Cells in the lower layer of the microporous membrane were fixed with 4% paraformaldehyde solution for 30 minutes and stained with 0.1% crystal violet staining solution at room temperature for 30 minutes. Five fields of view were randomly selected at 200× magnification to count the number of perforated cells, and their mean values were taken for statistical analysis.

### 2.7 Morphological observation of apoptosis

We observed and compared the apoptosis of each group (8 hours after normal culture or sugar deprivation) using an inverted microscope. The shrinking, rounding and falling off of cells were considered as the morphological characteristics of apoptosis, whereby the cells became smaller and deformed, and although the cell membrane was intact, foaming occurred.

### 2.8 siRNA transfection

The sequence of siRNA was used to express SIRT1 in the silent cells. SIRT1/FoxO1/Rab7-siRNA and negative control (NC)were purchased from Shanghai GenePharma Co., Ltd. The sequences of the siRNA are shown in **Table 1**. We used X-treme GENE(Switzerland Roche company) siRNA for transfer according to the operating instructions. The transfected cells were collected for subsequent experiments and the interference efficiency was detected by western blot.

**Table 1:** Sequence of siRNA.

Name of siRNA sequence	siRNA sequence
NC	sense primer: 5’-UUCUCCGAACGUGUCACGUTT-3’
	antisense primer: 5’-ACGUGACACGUUCGGAGAATT-3’
SIRT1-siRNA	sense primer: 5’-CCAGAGUCCAAGUUUAGAATT-3’
	antisense primer: 5’-UUCUAAACUUGGACUCUGGTT-3’
FoxO1-siRNA	sense primer: 5’-CCAUGGACAACAACAGUAATT-3’
	antisense primer: 5’-UUACUGUUGUUGUCCAUGGTT-3’
Rab7-siRNA	sense primer: 5’-CCAGACGAUUGCACGGAAUTT-3’
	antisense primer: 5’-AUUCCGUGCAAUCGUCUGGTT-3’

**Table 1** at the end of the manuscript.

### 2.9 mRFP-GFP-LC3 adenovirus infection to detect autophagy

Cells were inoculated in six-well plates at 2×10^5^ cells/well. Adenovirus infection can be performed when the degree of fusion reached about 50-70%. MRFP-GFP-LC3 adenovirus (Hanheng Biotechnology Co., Ltd.) infection was performed according to the operating instructions. The infected cells were collected, seeded, and used in intervention experiments (i.e., sugar deprivation and SIRT1 expression silencing). Autophagy was observed under a fluorescence microscope (fluorescence inverted phase contrast microscope, IX50, Olympus, Japan), and autophagic flux was assessed by counting the number of intracellular fluorescent spots (fluorescent spots/cell) of GFP and RFP.

### 2.10 Western blotting

The treated cells were washed twice with PBS and placed on ice with RIPA lysate for at least 30 min. The total protein of the cells was extracted and quantified. Cell lysates were added to 10% or 12% sodium dodecyl sulfate-polyacrylamide gel (SDS-PAGE) for separation and transferred to NC membranes, which were then sealed with 5% skim milk and incubated overnight with primary antibody at 4°C. The membrane was washed the next day with TBST and incubated with a secondary antibody for 1 hour at room temperature. Protein expression was detected by a chemiluminescence system (Millipore) according to the instructions.

### 2.11 Statistical analysis

Statistical analysis was performed using SPSS v13.0. Experimental data are expressed as the mean ± SD of three independent experiments. One-way ANOVA was used to compare multiple groups, and the LSD-t test was used for comparison between groups. Both P<0.05 and P<0.001 indicated a statistical difference.

## 3 Results

### 3.1 Cell proliferation of immortalized gastric epithelial cells and different GC cell lines under normal and GD conditions

**Figure 1:**
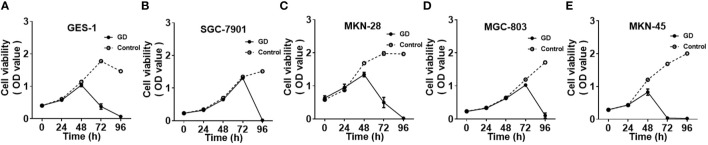
Cell growth and proliferation of different cell lines under GD and normal culture conditions; GES-1 **(A)**and MGC-803 **(D)** cells showed a significant decrease in proliferation after 72 hours under GD conditions (*P*<0.05 or *P*<0.001). MKN-28 **(C)** and MKN-45 **(E)** cells showed a significant decrease after 48 hours (*P*<0.05 or *P*<0.001). SGC-7901 **(B)**cells showed a significant decrease at 96 hours under GD conditions (*P*<0.001).

The cell growth and proliferation of gastric mucosal immortalized epithelial cells GES-1 and GC cells SGC-7901, MKN-28, MGC-803 and MKN-45 were detected by CCK-8 under normal and GD culture conditions. SGC-7901 had the longest tolerance time to GD (*P*<0.001) (**Figure 1B**), followed by GES-1(**Figure 1A**) and MGC-803 (**Figure 1D**)(*P*<0.05 or *P*<0.001). MKN-28 (**Figure 1C**) and MKN-45 (**Figure 1E**) cells were the least tolerant to GD(*P*<0.05 or *P*<0.001).

### 3.2 Expression of SIRT1 protein and autophagy-related protein in gastric mucosal immortalized epithelial cells and different gastric cancer cells

**Figure 2:**
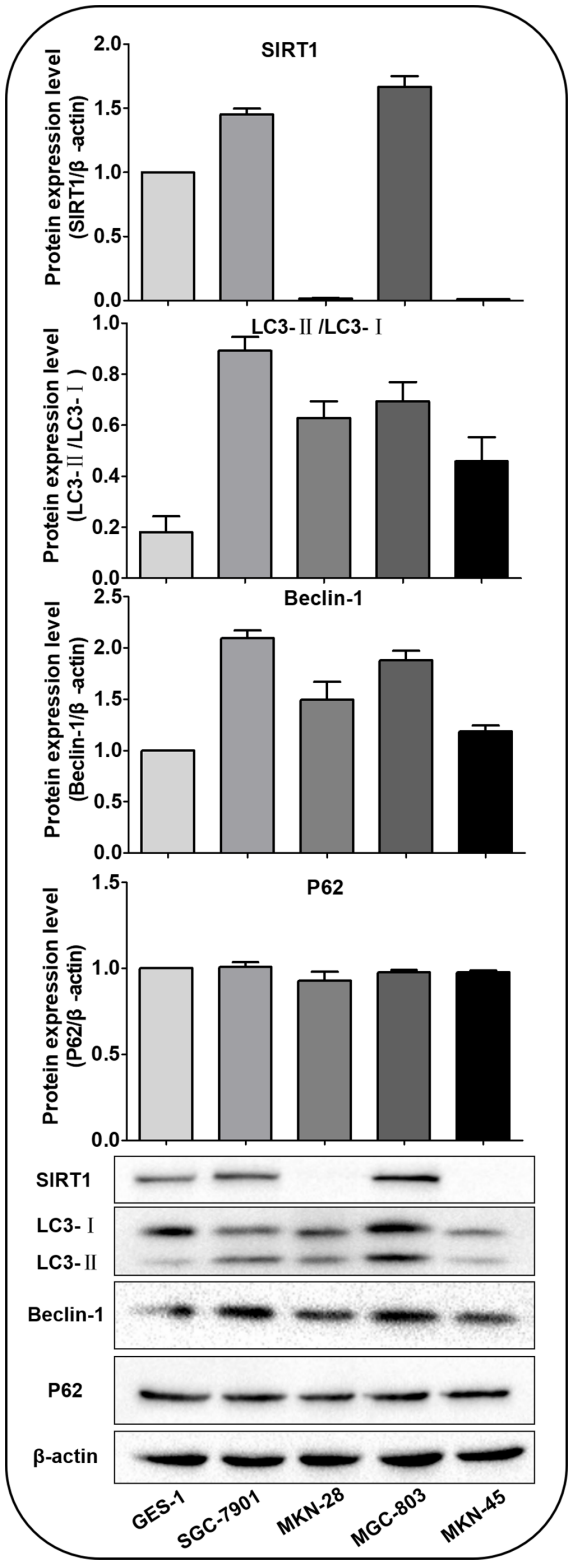
SIRT1 and autophagy-related proteins expressed in various cells under normal culture conditions. The expression of SIRT1 was higher in SGC-7901 and MGC-803 cells than in GES-1, MKN-45 and MKN-28 cells. The Beclin-1 and LC3-II/LC3-Iratio were highest in SGC-7901 cells. There was no significant difference in the expression of P62.

Western blot was performed to detect the expression of SIRT1 protein and autophagy-related proteins Beclin-1, LC3-II, LC3-I and P62 in GES-1, SGC-7901, MKN-28, MGC-803 and MKN-45 cells under normal culture conditions (**Figure 2**). The results showed that the highest level of basal autophagy was observed in SGC-7901 cells. Autophagy-associated protein (Beclin-1 and LC3-II/LC3-I ratio) was also correspondingly higher in SIRT1-high expressing cell lines, suggesting a connection between intracellular SIRT1 and autophagy. Therefore, we selected SGC-7901 cells, which demonstrated high expression of both SIRT1 and autophagy-related proteins for the subsequent experiments.

### 3.3 Intracellular autophagy of cells after incubation for different times under GD conditions

**Figure 3:**
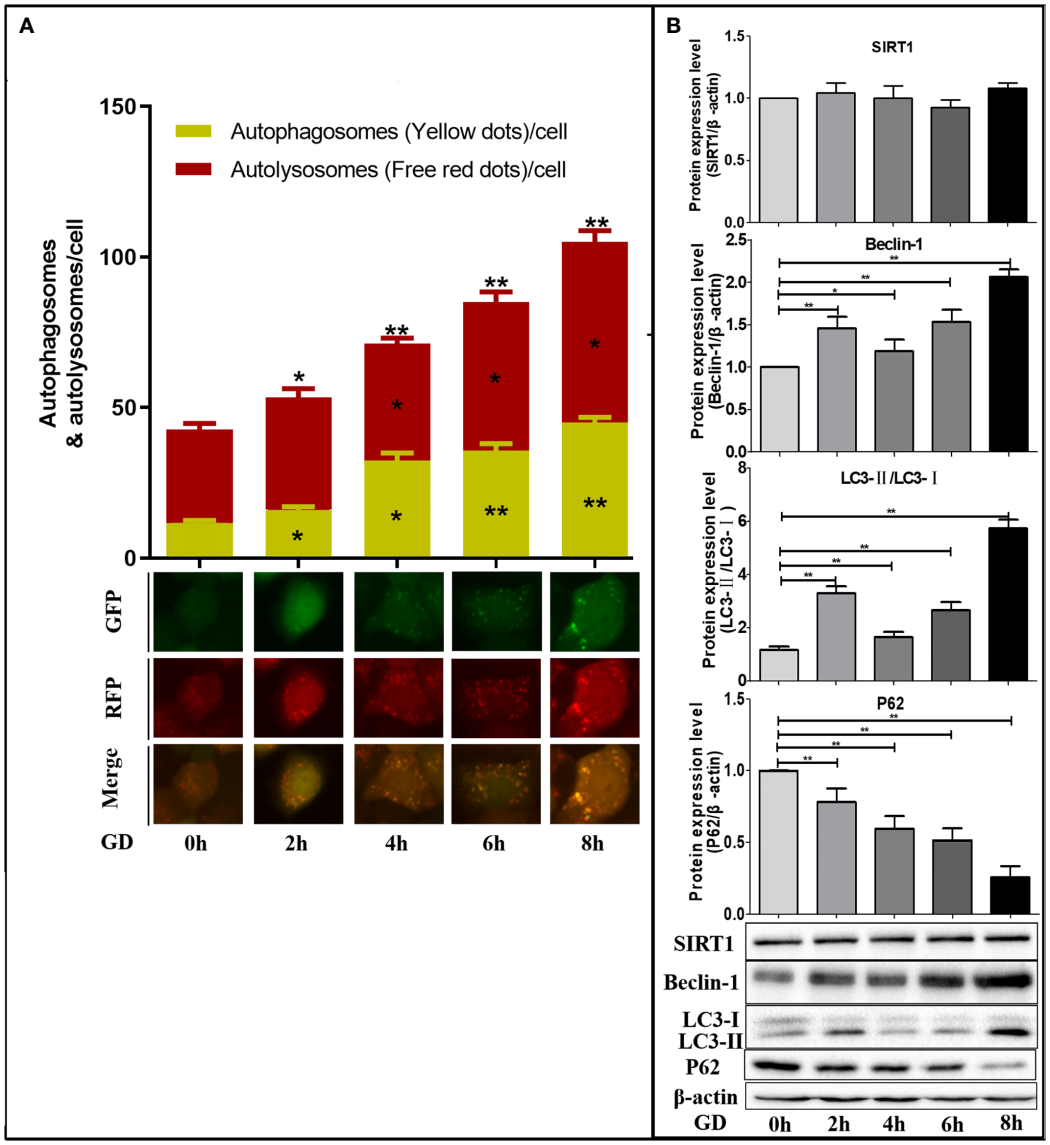
Autophagy in SGC-7901 cells after different times of GD. **(A)** Compared with GD at 0 hours, the number of yellow fluorescence (autophagosome), remaining red fluorescence (autophagolysosome) and total red fluorescence spots (total sum of autophagosome and autophagolysosome) increased gradually with GD time, and the differences were statistically significant (*P*<0.05 or *P*<0.001). **(B)** Western blot detected the expression of SIRT1 protein and autophagy-related protein (Beclin-1, LC3 and P62) after different times of GD culture. No significant changes in SIRT1 expression were observed, while Beclin-1 and LC3-II/LC3-I ratio significantly increased (*P*<0.05 or *P*<0.001), and P62 significantly decreased (*P*<0.05 or *P*<0.001). *P<0.05; **P<0.001.

SGC-7901 cells infected with mRFP-GFP-LC3 adenovirus for 48 hours were collected and inoculated. The cells were cultured in a sugar-free DMEM medium with 10% FBS for 24 hours. After 0, 2, 4, 6 and 8 hours, the cells were observed under an inverted fluorescent microscope, and the green, red and yellow fluorescence spots were compared after 0, 2, 4, 6 and 8 hours of incubation in DMEM medium without sugar 10% FBS (**Figure 3A**).

Compared with GD at 0 hours, the number of autophagosomes and autophagolysosomes was the highest at 8h of GD (**Figure 3A**). Therefore, intracellular autophagy activity gradually increased with the prolongation of GD time. Compared with GD at 0 hours, no significant changes in SIRT1 expression were observed after incubation with GD at 2, 4, 6 and 8 hours (**Figure 3B**). Comparatively, a significant increase in Beclin-1 and LC3-II/LC3-I ratio was observed (**Figure 3B**, *P*<0.05 or *P*<0.001), which were highest at 8 hours following GD. The expression of P62 was all significantly decreased (**Figure 3B**, *P*<0.05 or *P*<0.001) and lowest at 8 hours of GD.

Since the intracellular autophagy level was the highest after GD culture for 8 hours, we selected GD culture for 8 hours to detect the effect and mechanism of SIRT1 on the autophagy of GC cells under GD condition.

### 3.4 Interrelationship between SIRT1, FoxO1 and Rab7 in SGC-7901 cells under GD conditions

**Figure 4:**
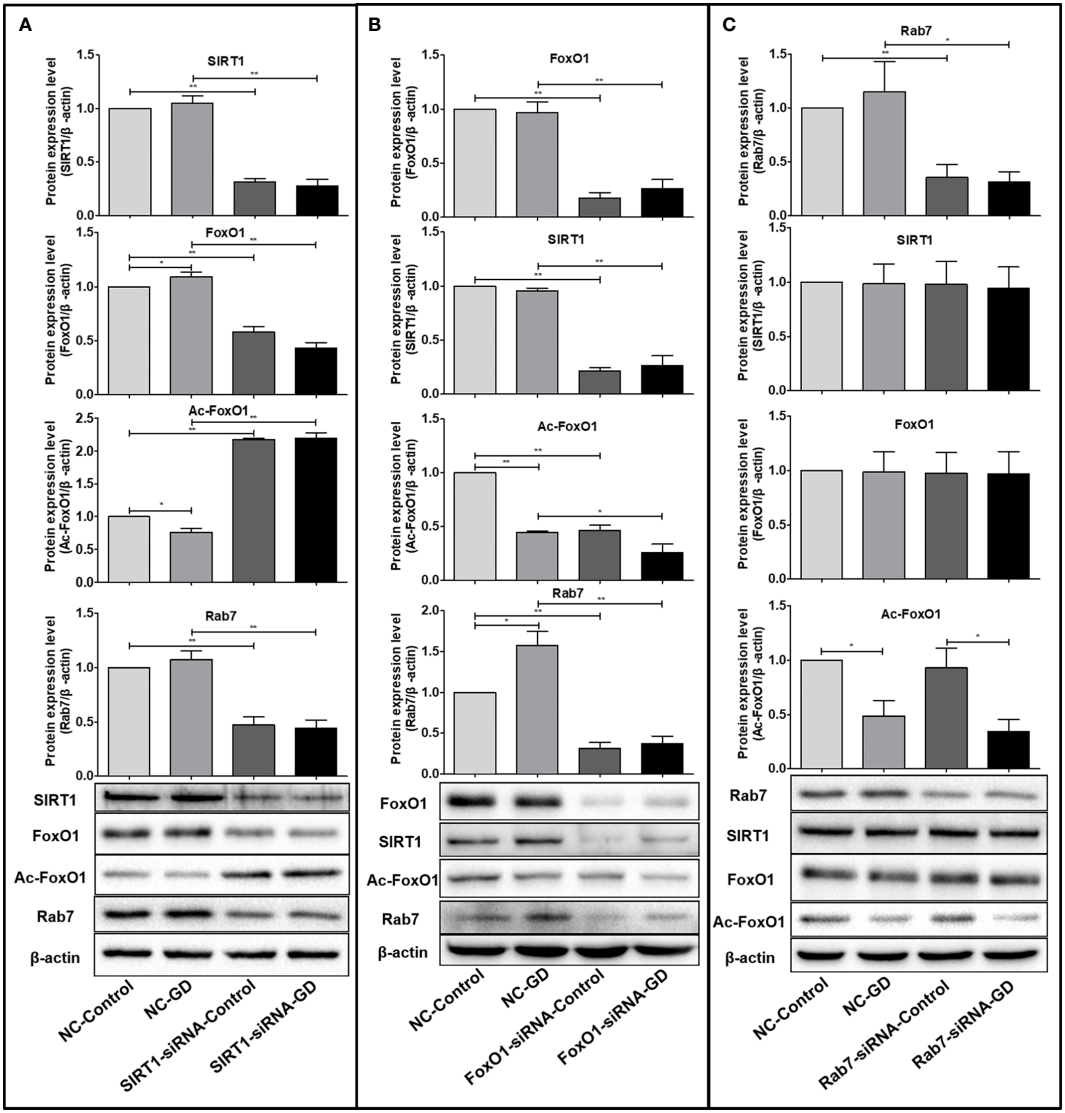
Relationship between SIRT1, FoxO1 and Rab7 protein expression. **(A)** FoxO1 and Rab7 protein expression were significantly lower in the SIRT1-siRNA-Control and SIRT1-siRNA-GD groups compared with the NC-Control and NC-GD groups (*P*<0.05), while the expression of Ac-FoxO1 was opposite. (*P*<0.05). **(B)** The expression of SIRT1, Ac-FoxO1 and Rab7 in the FoxO1-siRNA-Control and FoxO1-siRNA-GD groups were significantly lower than in the NC-Control and NC-GD groups (*P*<0.05 or *P*<0.001). **(C)** The expression of SIRT1, FoxO1 and Ac-FoxO1 in the Rab7-siRNA-Control and Rab7-siRNA-GD groups did not change significantly compared with the NC-Control and NC-GD groups, respectively (*P*>0.05). *P<0.05; **P<0.001.

The SIRT1(FoxO1)(Rab7)protein expression in cells of SIRT1(FoxO1)(Rab7)-siRNA-Control/SIRT1(FoxO1)(Rab7)-siRNA-GD group was significantly lower than those in the NC-Control/NC-GD group, respectively (*P*<0.001, **Figure 4**), indicating that SIRT1(FoxO1)(Rab7)-siRNA can effectively reduce intracellular SIRT1(FoxO1)(Rab7) expression.

FoxO1 and Rab7 protein expression were significantly lower in SIRT1-siRNA-Control and SIRT1-siRNA-GD groups compared with NC-Control and NC-GD groups, respectively (**Figure 4A**, *P*<0.001), while opposite results were obtained for the expression of Ac-FoxO1 (**Figure 4A**, *P*<0.001). This result indicated that reducing intracellular SIRT1 expression could significantly reduce intracellular FoxO1 and Rab7 expression under normal and GD conditions while leading to significantly higher intracellular Ac-FoxO1 levels. The SIRT1, Ac-FoxO1 and Rab7 expression in the FoxO1-siRNA-Control and FoxO1-siRNA-GD groups were significantly lower than in the NC-Control and NC-GD groups (**Figure 4B**, *P*<0.05 or *P*<0.001), which indicated that the decreased expression of FoxO1 could affect the expression of SIRT1, Ac-FoxO1 and Rab7 in cells. The expression of SIRT1, FoxO1 and Ac-FoxO1 in the Rab7-siRNA-Control and Rab7-siRNA-GD groups did not change significantly compared with the groups NC-Control and NC-GD groups, respectively(**Figure 4C**, *P*>0.05). The results showed that the decrease in intracellular Rab7 did not affect the expression of SIRT1, FoxO1 and Ac-FoxO1 under normal and GD conditions.

Combined with the above results, we found that under GD conditions, cells regulated the deacetylation modification of Ac-FoxO1 by SIRT1, which in turn affected the expression of FoxO1 and Rab7. In addition, the expression of FoxO1 could act as a positive feedback regulation to promote SIRT1 expression.

### 3.5 The SIRT1-FoxO1-Rab7 axis regulates SGC-7901 cells autophagy under GD conditions

Here, we investigated the autophagy levels in SGC-7901 cells with normal expression (NC group) and reduced expression (SIRT1/FoxO1/Rab7-siRNA group) of SIRT1, FoxO1 and Rab7 under normal and 8 hours of GD culture conditions.

**Figure 5:**
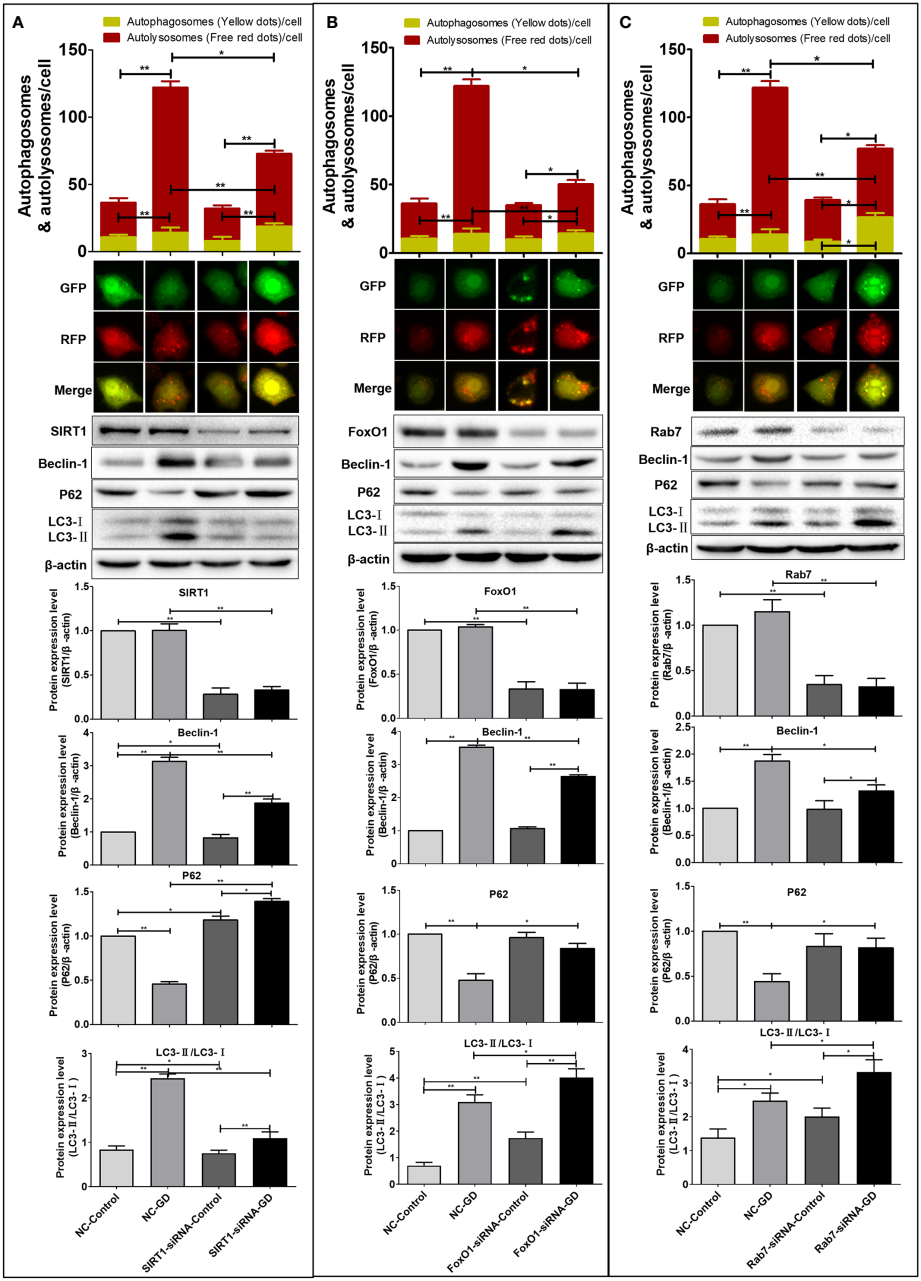
Effects of SIRT1, FoxO1 and Rab7 on autophagy in SGC-7901 cells under GD condition. **(A)** The number of remaining red fluorescent spots and total fluorescent spots in cells of the SIRT1-siRNA-GD was greater than those in the SIRT1-siRNA-Control (*P*<0.001). The Beclin-1, LC3-II/LC3-I ratio and P62 expression in the SIRT1-siRNA-Control, SIRT1-siRNA-GD, NC-Control and NC-GD groups. **(B)** The number of remaining red fluorescent spots and total fluorescent spots in cells of FoxO1-siRNA-GD was greater than those in FoxO1-siRNA-Control (*P*<0.001). The Beclin-1, LC3-II/LC3-I ratio and P62 in the FoxO1-siRNA-Control, FoxO1-siRNA-GD, NC-Control and NC-GD groups. **(C)** The number of yellow fluorescent spots, remaining red fluorescent spots and total fluorescent spots in cells of the Rab7-siRNA-GD group were greater than those in the Rab7-siRNA-Control group (*P*<0.05). The Beclin-1, LC3-II/LC3-I ratio and P62 in the Rab7-siRNA-Control, Rab7-siRNA-GD, NC-Control and NC-GD groups. *P<0.05; **P<0.001.

Compared with the NC-Control group, the number of remaining red fluorescent spots (*P*<0.001) and total fluorescent spots (*P*<0.001) in the NC-GD group were significantly increased (**Figure 5**). The number of residual red fluorescent spots (*P*<0.001) and total fluorescent spots (*P*<0.001) in the SIRT1-siRNA-GD and FoxO1-siRNA-GD groups were also greater than those in the SIRT1-siRNA-Control and FoxO1-siRNA-Control groups, respectively (**Figures 5A, B**). The number of yellow, remaining red and total fluorescent spots in the Rab7-siRNA-GD group were greater than those in the Rab7-siRNA-Control group (*P*<0.05), which showed that 8 hours of GD could increase the level of autophagy (**Figure 5**). The number of intracellular yellow, red and total fluorescent spots in the SIRT1-siRNA-Control, FoxO1-siRNA-Control and Rab7-siRNA-Control groups were not significantly greater than those in the NC-Control group (*P*>0.05). However, although the intracellular yellow fluorescent spots in the SIRT1-siRNA-GD, FoxO1-siRNA-GD and Rab7-siRNA-GD groups were more than those in the NC-GD group, the difference was not statistically significant (*P*>0.05). The number of red fluorescent spots and total fluorescent spots were significantly less than those in the NC-GD group (**Figure 5**, *P*<0.05 or *P*<0.001). Therefore, reducing the expression of intracellular SIRT1, FoxO1 or Rab7 did not affect intracellular autophagy under normal culture conditions but significantly inhibited intracellular autophagy under GD conditions.

Western blot was used to detect the effect of SIRT1, FoxO1 or Rab7 on the expression of autophagy-related proteins (Beclin-1, LC3 and P62). The results showed that compared with NC-Control, SIRT1-siRNA-Control, FoxO1-siRNA-Control and Rab7-siRNA-Control, the intracellular LC3-II/LC3-I ratio and Beclin-1 expression in the NC-GD, SIRT1-siRNA-GD, FoxO1-siRNA-GD and Rab7-siRNA-GD groups were significantly increased (**Figure 5**, *P*<0.05 or *P*<0.001). The expression of P62 in the NC-GD group was significantly lower than that in the NC-Control group(*P*<0.001) but significantly higher in the SIRT1-siRNA-GD group than the SIRT1-siRNA-Control group (*P*<0.05). However, there was no significant difference in the expression of P62 in the FoxO1-siRNA-GD and Rab7-siRNA-GD groups compared with the FoxO1-siRNA-Control and Rab7-siRNA-Control groups, respectively (*P*>0.05).

Therefore, GD could increase the autophagy in cells with normal or reduced expression of SIRT1, FoxO1 or Rab7. However, reduced expression of SIRT1, FoxO1 and Rab7 blocked the intracellular autophagic flow (**Figure 5**). Compared with NC-Control and NC-GD, respectively, the LC3-II/LC3-I and Beclin-1 of cells in the SIRT1-siRNA-Control and SIRT1-siRNA-GD groups were significantly decreased (**Figure 5A**, *P*<0.05 or *P*<0.001), while P62 was significantly increased (**Figure 5A**, *P*<0.05 or *P*<0.001). The ratio of LC3-II/LC3-I in the FoxO1-siRNA-Control, FoxO1-siRNA-GD, Rab7-siRNA-Control and Rab7-siRNA-GD groups was significantly increased (**Figures 5B, C**, *P*<0.05 or *P*<0.001). There was no significant difference in the expression of Beclin-1 and P62 in the FoxO1-siRNA-Control and Rab7-siRNA-Control group compared to the NC-Control group (**Figures 5B, C**, *P*>0.05). The expression of Beclin-1 in the FoxO1-siRNA-GD and Rab7-siRNA-GD groups was significantly lower than in NC-GD, while P62 expression was significantly increased (**Figure 5B, C**, *P*<0.05 or *P*<0.001). Therefore, reducing the expression of intracellular SIRT1, FoxO1 or Rab7 could significantly inhibit the flow of autophagy in cells under GD conditions, indicating that SIRT1 could affect autophagy in GC cells through the regulation of Rab7 by FoxO1.

### 3.6 The SIRT1-FoxO1-Rab7 axis regulates SGC-7901 cells proliferation under GD conditions

**Figure 6:**
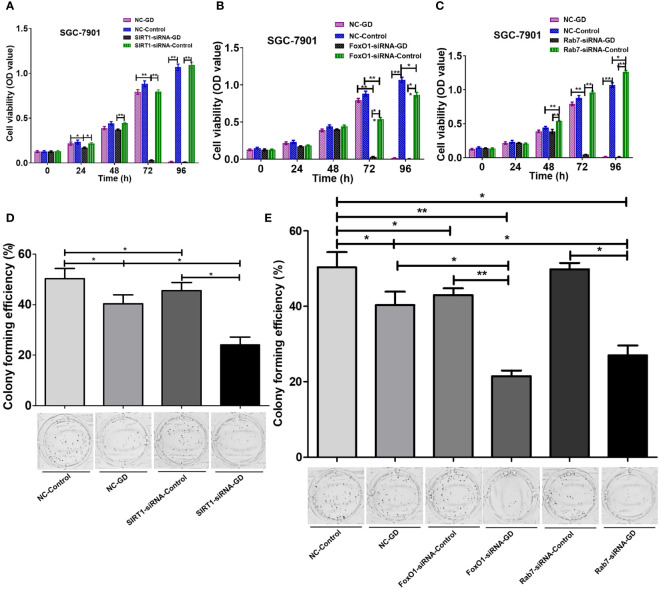
Effects of SIRT1-FoxO1-Rab7 axis on the cell proliferation of SGC-7901 cells under GD conditions. **(A)** No significant difference in the proliferation of cells in SIRT1-siRNA-Control compared to NC-Control (*P*>0.05). The proliferation of SIRT1-siRNA-GD was significantly lower at 72 hours (*P*<0.001). **(B)** FoxO1-siRNA-Control showed reduced proliferation at 72 and 96 hours of culture compared to NC-Control (*P*<0.05 or *P*<0.001). The proliferation of FoxO1-siRNA-GD was significantly lower at 72 hours (*P*<0.001). **(C)** Rab7-siRNA-Control showed enhanced proliferation at 48 hours compared to NC-Control (*P*<0.001). The proliferation of Rab7-siRNA-GD was significantly lower at 72 hours (*P*<0.001). **(D, E)** The clone formation rates of NC-GD, SIRT1-siRNA-GD, FoxO1-siRNA-GD and Rab7-siRNA-GD were significantly reduced (*P*<0.05 or *P*<0.001). *P<0.05; **P<0.001.

We investigated the effect of the SIRT1-FoxO1-RAB7 axis on the proliferation of GC SGC-7901 cells by CCK-8 and plate clone formation assays. The results showed no significant difference between the proliferation of cells in the SIRT1-siRNA-Control group and the NC-Control group (**Figure 6A**, *P*>0.05). Cells in the FoxO1-siRNA-Control group had reduced proliferation at 72 and 96 hours of culture (**Figure 6B**, *P*<0.05 or *P*<0.001), while cells in the Rab7-siRNA-Control group had enhanced proliferation at 48 hours (*P*<0.001), 72 hours(*P*>0.05) and 96 hours (*P*<0.001)(**Figure 6C**). However, cells in the NC-GD group had reduced proliferation at 96 hours of culture under GD conditions (**Figures 6A-C**, *P*<0.001). Thus, intracellular SIRT1 expression did not affect cell proliferation under normal culture conditions. Additionally, we observed that a decrease in FoxO1 expression inhibited proliferation under normal culture conditions, and reduced Rab7 expression promoted the proliferation of GC cells under normalconditionsn, while GD inhibited the proliferation. SIRT1-siRNA-GD, FoxO1-siRNA-GD and Rab7-siRNA-GD showed a significant decrease in cell proliferation at 24, 48 and 72 hours of GD culture compared with SIRT1-siRNA-Control, FoxO1-siRNA-Control and Rab7-siRNA-Control. Furthermore, the inhibition was more evident with time (**Figures 6A-C**, *P*<0.05 or *P*<0.001). Compared with NC-GD, the proliferation of cells in the SIRT1-siRNA-GD group was lower at 24 hours under GD conditions (**Figure 6A**, *P*<0.05), and the difference was more significant at 72 hours (**Figure 6A**, *P*<0.001). The proliferation of cells in FoxO1-siRNA-GD and Rab7-siRNA-GD was significantly lower at 72 hours under GD conditions (**Figures 6B, C** *P*<0.001).

The results of the plate cloning experiment (**Figures 6D, E**) showed that compared with NC-Control, SIRT1-siRNA-Contral, FoxO1-siRNA-Control and Rab7-siRNA-Control, the clone formation rates of NC-GD, SIRT1-siRNA-GD, FoxO1-siRNA-GD and Rab7-siRNA-GD were significantly reduced (*P*<0.05 or *P*<0.001). The results showed that GD could decrease the proliferation ability of the cells during normal and reduced expression of SIRT1, FoxO1 and Rab7. Clone formation rates were also lower in the SIRT1-siRNA-Control/FoxO1-siRNA-Control and SIRT1-siRNA-GD/FoxO1-siRNA-GD groups, compared to groups the NC-Control and NC-GD groups, respectively (*P*<0.05). Comparatively, the differences between clone formation rates in the Rab7-siRNA-Control and NC-Control groups were not statistically significant (*P*>0.05), and the clone formation rate in the Rab7-siRNA-GD group was significantly lower than in the NC-GD group (*P*<0.05). Thus, decreased SIRT1, FoxO1 or Rab7 expression could enhance the inhibitory effects of GD on cell proliferation.

### 3.7 The SIRT1-FoxO1-Rab7 axis regulates SGC-7901 cells migration and invasion under GD condition

**Figure 7:**
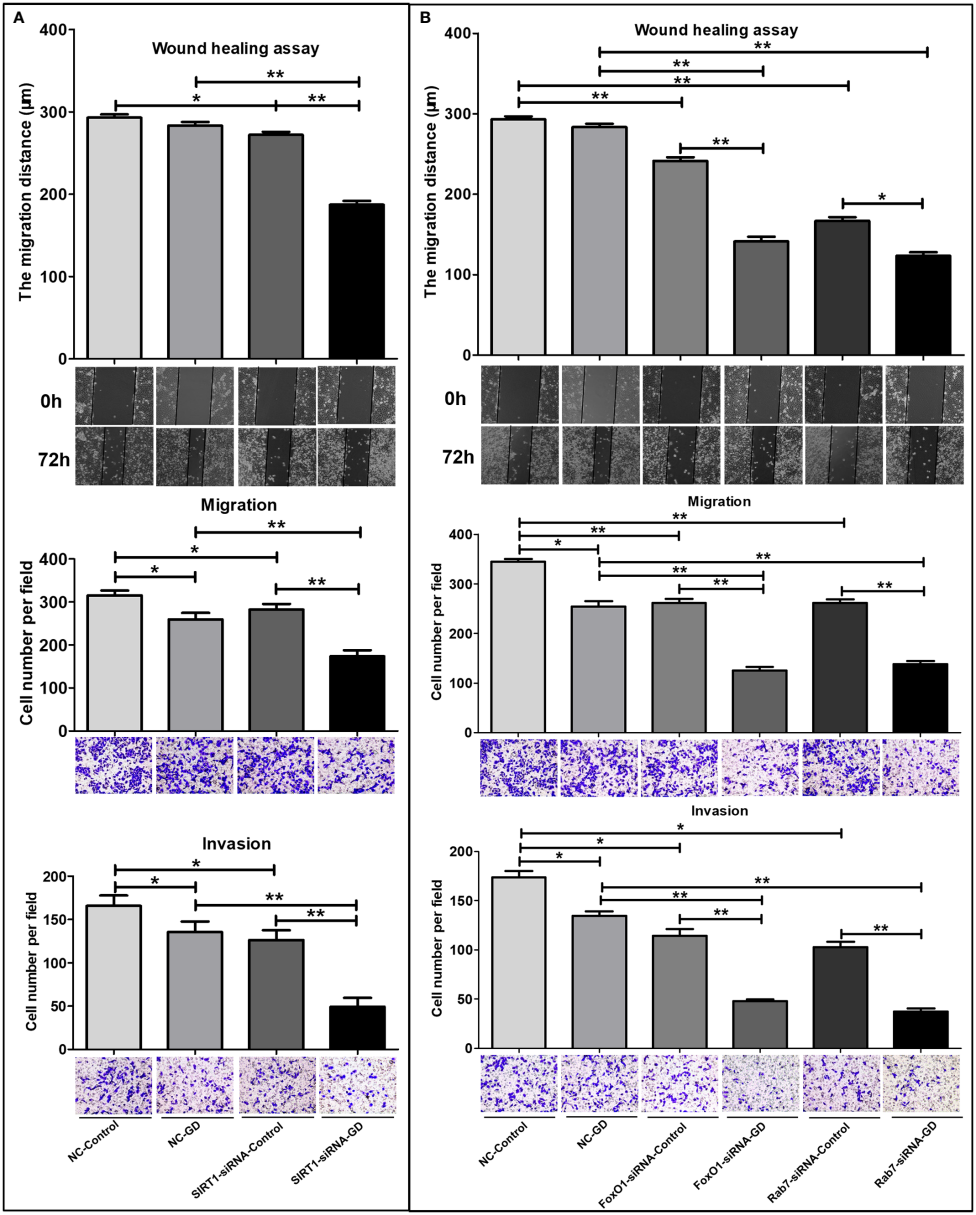
Effects of SIRT1, FoxO1, Rab7 on the cell migration and invasion of SGC-7901 cells under GD condition. **(A)** The relative migration distance and the number of transmembrane cells of the SIRT1-siRNA-GD and SIRT1-siRNA-Control groups. **(B)** The relative migration distance and the number of transmembrane cells of FoxO1-siRNA-GD, Rab7-siRNA-GD, FoxO1-siRNA-Control and Rab7-siRNA-Control groups. *P<0.05; **P<0.001.

Scratch tests and Transwell experiments were performed to investigate the effects of the SIRT1-FoxO1-Rab7 axis on cell migration and invasion in SGC-7901 cells under GD. The results showed that the relative migration distance of cells in NC-GD was not significantly different at 72 hours after scratching compared with the NC-Control group (**Figure 7A**, *P*>0.05). The relative migration distance of SIRT1-siRNA-GD, FoxO1-siRNA-GD and Rab7-siRNA-GD at 72 hours after scratching was significantly lower than that of the SIRT1-siRNA-Control (**Figure 7A**, *P*<0.001), FoxO1-siRNA-Control and Rab7-siRNA-Control groups (**Figure 7B**, *P*<0.001); respectively. Therefore, GD did not affect the migration of cells with normal expression of SIRT1, FoxO1 and Rab7 but significantly inhibited the migration of cells with low expression of SIRT1, FoxO1 or Rab7. Compared with NC-Control, the relative migration distances of cells in SIRT1-siRNA-Control, FoxO1-siRNA-Control and Rab7-siRNA-Control were shorter at 72 hours after scratching (**Figures 7A, B**, *P*<0.05 or *P*<0.001). Compared with NC-GD, the relative migration distances of cells in the SIRT1-siRNA-GD, FoxO1-siRNA-GD and Rab7-siRNA-GD groups were also shorter at 72 hours after scratching (**Figure 7A, B**, *P*<0.001).

Transwell migration and invasion assay showed that the number of transmembrane cells in the NC-GD, SIRT1-siRNA-GD, FoxO1-siRNA-GD and Rab7-siRNA-GD groups were lesser than those in the NC-Control, SIRT1-siRNA-Control, FoxO1-siRNA-Control and Rab7-siRNA-Control groups (**Figure 7**, *P*<0.05 or *P*<0.001), respectively. This indicated that GD could inhibit the migration and invasion of GC cells with normal or low expression of SIRT1, FoxO1 and Rab7. The number of transmembrane cells in the SIRT1-siRNA-Control, FoxO1-siRNA-Control and Rab7-siRNA-Control groups were lower than in the NC-Control group(*P*<0.05 or *P*<0.001). SIRT1-siRNA-GD, FoxO1-siRNA-GD and Rab7-siRNA-GD all had fewer transmembrane cells (*P*<0.001) than NC-GD, respectively, suggesting that reduced SIRT1, FoxO1 or Rab7 expression could significantly reduce the ability of cell migration and invasion during GD.

### 3.8 The SIRT1-FoxO1-Rab7 axis regulates SGC-7901 cells apoptosis under GD condition

**Figure 8:**
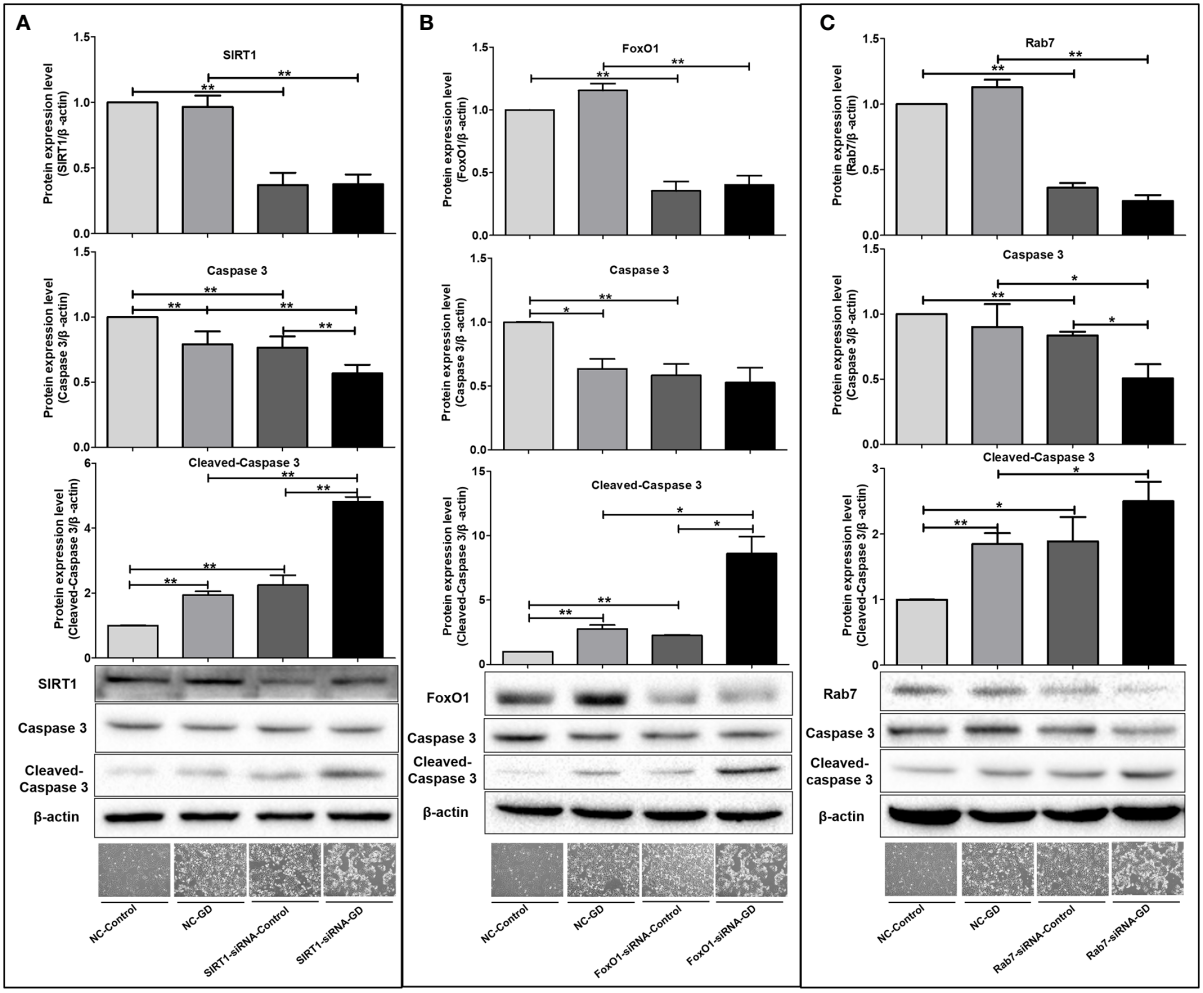
Effects of SIRT1, FoxO1 and Rab7 on cell apoptosis in SGC-7901 cells under GD condition. **(A)** The shrunken, rounded and shed cells and the expression of intracellular Caspase 3 and Cleaved-Caspase 3 in the SIRT1-siRNA-Control and SIRT1-siRNA-GD groups. **(B)** The shrunken, rounded and shed cells and the expression of intracellular Caspase 3 and Cleaved-Caspase 3 in the FoxO1-siRNA-Control and FoxO1-siRNA-GD groups. **(C)** The shrunken, rounded and shed cells and the expression of intracellular Caspase 3 and Cleaved-Caspase 3 in the Rab7-siRNA-Control and Rab7-siRNA-GD groups. *P<0.05; **P<0.001.

Observation under an inverted microscope (**Figure 8**) showed that the shrunken, rounded and shed cells in the visual fields of the NC-GD, SIRT1-siRNA-Control, FoxO1-siRNA-Control and Rab7-siRNA-Control groups were significantly greater than the NC-Control group, but significantly lesser than those in the SIRT1-siRNA-GD, FoxO1-siRNA-GD and Rab7-siRNA-GD groups.

Next, we examined the intracellular apoptosis-related proteins in each group and found that the expression of intracellular Cleaved-Caspase 3 was significantly higher in the NC-GD, SIRT1-siRNA-GD, FoxO1-siRNA-GD and Rab7-siRNA-GD groups compared with the NC-Control, SIRT1-siRNA-Control, FoxO1-siRNA-Control and Rab7-siRNA-Control groups (**Figure 8**, *P*<0.001), suggesting that GD increased apoptosis in cells with normal or decreased SIRT1, FoxO1 and Rab7 expression. The level of Caspase 3 in the SIRT1-siRNA-Control, FoxO1-siRNA-Control and Rab7-siRNA-Control groups was significantly lower than the NC-Control group, while opposite results were observed for the expression of Cleaved-Caspase 3 (**Figure 8**, *P*<0.001). Caspase 3 in SIRT1-siRNA-GD, FoxO1-siRNA-GD and Rab7-siRNA-GD cells was also significantly lower than in NC-GD cells, while Cleaved-Caspase 3 expression was significantly increased (**Figure 8**, *P*<0.001). In addition, intracellular Caspase 3 was the lowest in SIRT1-siRNA-GD, FoxO1-siRNA-GD and Rab7-siRNA-GD cells, while the expression of Cleaved-Caspase 3 was highest. Therefore, decreasing the expression of SIRT1, FoxO1 or Rab7 could significantly increase apoptosis under GD conditions.

## 4 Discussion

Current studies have reported a high expression of SIRT1 in GC tissues and association with advanced lymph node metastasis (20). Moreover, SIRT1 was shown to regulate EMT and the invasive ability of GC cells (21). SIRT1-mediated autophagy also plays a critical role in cell proliferation, metabolism and stress reaction (4–6, 22). Therefore, SIRT1 is not only involved in the development of GC and its therapeutic response but can also be considered one of the crucial molecules regulating autophagy. Although several studies have identified SIRT1 as a key molecule in the development of intracellular autophagy under GD or nutrient-deprivation conditions (23–25), the specific mechanisms were different. Therefore, based on the types of GC cells and the environments in which they are located, the mechanisms of SIRT1 that influence the malignant biological behavior of GC cells under GD may also differ from many previous studies.

It was found that GD could activate intracellular autophagic activity *via* multiple signaling pathways (26). Fu Z et al. (27) found that the protection mechanism of Dex on brain ischemia-reperfusion (I/R) injury might relate to the activation of SIRT3-mediated autophagy. Walker et al. (28) concluded that Nrf2 signaling *via* its antioxidant activity was critical in protecting cells during GD-induced autophagy. Thus, increased intracellular autophagic activity under GD promotes cell resistance to the adverse environment. However, the activity and role of autophagy in GC cells under GD conditions remained unclear. Thus, we designed this present study to investigate the growth, proliferation, migration, invasion, apoptosis and autophagic activity of GC cells under GD. Our results indicated that normal gastric mucosa immortalized epithelial cells GES-1 and GC cell lines showed different cell growth and proliferation at different GD durations. SGC-7901 cells had the longest toleration time to GD (>72 hours), followed by GES-1 and MGC-803 (48-72 hours), while MKN-28 and MKN-45 (<48 hours) had the least toleration time. Therefore, the level of autophagy within GC cells under GD conditions affected cell survival and growth.

Our previous study found that the expression of Beclin-1 and SIRT1 in GC tissues was higher than in corresponding adjacent normal gastric mucosa (7). The expression of Beclin-1 in GC tissues was significantly and positively correlated with the expression of SIRT1. Patients with high Beclin-1, SIRT1 and Beclin-1/SIRT1 expression had shorter OS and RFS. These results suggested that SIRT1 could promote GC progression by regulating autophagy. Further, SGC-7901 cells were used for further expermental analysis. We created GD conditions to simulate the glucose-deficient environment in GC to explore whether SIRT1 affected the autophagy activity, cell proliferation, migration, invasion and apoptosis of GC cells. The results showed that reduced SIRT1 expression significantly reduced the autophagy activity of GC cells under GD conditions. It further suggested a close association between SIRT1 and autophagy occurrence in GC cells under GD conditions. Recently, many studies have also demonstrated SIRT1 as a key molecule for the occurrence of autophagy under starvation conditions. Wang et al. (29) found that MALAT1 activated autophagy by binding to miR-200c-3p and upregulating SIRT1 expression to protecte BMECs (brain microvascular endothelial cells) against oxygen-GD injury. Cao et al. (30) proposed a novel mechanism by which PCA alleviates oxygen-GD injury to HUVECs (human umbilical vein endothelial cells) by promoting autophagy and inhibiting apoptosis through the SIRT1 axis. Liu et al. (31) indicated that melatonin could protect against cerebral ischemia-reperfusion (CIR)-induced brain damage in diabetic mice, which was achieved by the autophagy enhancement mediated by the SIRT1-BMAL1 pathway. Therefore, SIRT1 can be considered as a key molecule for intracellular autophagy, especially under GD or starvation conditions.

Previous studies have found that directly or indirectly reducing or increasing the expression of SIRT1 in tumor cells significantly impacted the cells’biological behavior. SIRT1 was shown to be a target of microRNA-12129 (32). SIRT1 expression was negatively related to microRNA-12129, which can suppress cell proliferation and cell cycle progression in GC by targeting SIRT1. Both *in vivo* and *in vitro* studies showed that SIRT1 overexpression could promote the migration and invasion of colorectal cancer cells while reducing SIRT1 expression inhibited the migration and invasion of the cells (33). Yarahmadi et al. (34) reported that SIRT1 expression was a risk factor for breast cancer, and inhibiting its expression significantly inhibited cancer cell invasion. In pancreatic cancer, SIRT1 was reported to promote proliferation, autophagy and invasion. Thus, the above studies showed that SIRT1 could promote the proliferation, migration and invasion of tumor cells and inhibit apoptosis. Although our results showed that GD alone could slightly inhibit the migration and invasion of GC SGC-7901 cells and mildly promote cell apoptosis, the decrease in SIRT1 expression inhibited the intracellular autophagy activity under GD condition, significantly reduced the tolerance of GC cells to GD, enhanced the inhibition of GC cell proliferation, migration, invasion and apoptosis by GD. On the contrary, some studies have reported that SIRT1 acted as a tumor suppressor as it inhibited the proliferation, migration and invasion of tumor cells and promoted apoptosis and cycle arrest (35, 36), thereby indicating that the effect of SIRT1 on GC or other tumors could be closely related to the specific environment and tumor type. Therefore, comprehensive considerations and settings are required when studying the effects of SIRT1 on GC.

The study found that under starvation conditions, SIRT1 could deacetylate FoxO1 and further upregulate the expression of Rab7 (a small GTP-binding protein that mediates autophagosome-lysosome fusion) to increase the level of autophagy in cardiomyocytes, to help cardiomyocytes resist the effects of starvation (9). In this study, we found that reducing the expression of SIRT1 in GC cells could significantly reduce the expression of FoxO1 and Rab7 in cells and increase the level of Ac-FoxO1, indicating that FoxO1 and Rab7 might be the downstream molecules of SIRT1, which can deacetylate FoxO1 and affect its gene transcription function. After using siRNA to reduce FoxO1’s expression levels, Ac-FoxO1, SIRT1 and Rab7 were inhibited. However, the inhibition of SIRT1 expression did not increase intracellular Ac-FoxO1, suggesting that Ac-FoxO1 was mainly increased with total intracellular FoxO1 level and that Rab7 expression was controlled by FoxO1. Reducing the expression of intracellular Rab7 did not significantly affect the changes in intracellular SIRT1, FoxO1 and Ac-FoxO1, indicating that Rab7 might also be located downstream of SIRT1 and FoxO1.

Hariharan et al. (9) found that GD could up-regulate the expression of SIRT1 in cardiomyocytes, and then deacetylate FoxO1 and promote nuclear transcription and protein activation, thereby increasing the expression of Rab7 and finally activating intracellular autophagy; under GD conditions, Rab7 overexpression enhanced autophagy in these cells; in contrast, Rab7 or FoxO1 knockout or FoxO1 mutation inhibited autophagy. Therefore, these findings suggested that GD could induce autophagy through the SIRT1-FoxO1-Rab7 axis. Our findings in gastric cancer cells are consistent with this. Other studies have also found that long-term administration of resveratrol increase autophagy levels by promoting SIRT1 activity and up-regulating Rab7 expression, thereby improving oxidative damage in the hearts of diabetic mice (37); However, inhibition of autophagy did not affect the activity of intracellular SIRT1 or the expression level of Rab7 (37); These results indicated that SIRT1 and Rab7 can affect autophagy in cells under the condition of glucose deficiency, but autophagy cannot affect their expression. At the same time, administration of resveratrol reversed oxidative stress damage to H9C2 cells and enhanced the binding of FoxO1 DNA to the Rab7 promoter region in a SIRT1-dependent manner, promoting the expression of Rab7 (37). As the main regulator of the fusion process of autophagosome and lysosome, Rab7 is essential for the successful completion of autophagy process (38). Therefore, these findings suggest the role of the SIRT1-FoxO1-Rab7 axis in the occurrence of intracellular autophagy under glucose-starved conditions.

To further investigate whether SIRT1 affects the malignant biological behavior of gastric cancer cells under GD conditions through FoxO1-Rab7-autophagy, we used siRNA interference technology to artificially reduce the expression of FoxO1 or Rab7 in cells. We found that the decrease in intracellular FoxO1 (Rab7) expression could significantly inhibit the level of autophagy in SGC-7901 cells under GD conditions. A decrease in intracellular FoxO1 (Rab7) expression could reduce the tolerance of GC cells to GD, inhibit the proliferation, migration and invasion of GC cells under GD conditions. These results further indicated that SIRT1 affected the malignant biological behavior of GC cells under GD conditions through FoxO1-Rab7-autophagy.

It was previously reported that decreased FoxO1 expression could inhibit tumor formation, while an increase in its expression promoted tumor resistance to drugs (39). In GC, phosphorylated FoxO1 plays an important role in tumor angiogenesis (40). In addition, FoxO1 can also regulate GC cell resistance to cisplatin and inhibit the apoptosis of GC cells by activating the PI3K/Akt signaling pathway (41). Therefore, different post-transcriptional modifications of FoxO1 play different roles in the progression of GC. In this study, we found that under GD, reducing the expression of FoxO1 in GC cells by siRNA significantly inhibited cell autophagy, proliferation, migration and invasion, suggesting that FoxO1 could promote GC progression in a glucose-deficient environment and was involved in GC cell autophagy.

Cellular functional experiments showed that the overexpression of Rab7 induced transition from S phase to G2 phase and promoted the proliferation, invasion and migration of GC cells (42). In this present study, we found that under GD, reducing intracellular SIRT1 or FoxO1 significantly inhibited the expression of Rab7 and the level of intracellular autophagy. Therefore, these results suggested that GC cells under GD conditions could affect the level of intracellular autophagy *via* the SIRT1-FoxO1-Rab7 pathway.

However, in studying the effect of the SIRT1-FoxO1-Rab7 axis on the autophagy and malignant biological behavior of GC cells under GD conditions, we only used siRNA interference technology to reduce SIRT1, FoxO1 and Rab7 proteins in GC cells with high SIRT1 expression and did not treat the cells with SIRT1, FoxO1 or Rab7 inhibitors to verify their effects on the cells. In addition, we did not perform the same study using SIRT1 low-expressing GC cells and SIRT1, FoxO1 or Rab7 agonists to validate the results. Therefore, more researches are needed to validate our findings.

Currently, there has been no research on the effect and mechanism of SIRT1 on autophagy and the malignant biological behavior of GC cells under GD conditions. This study is the first to unveil SIRT1-FoxO1-Rab7 as a key pathway for the normal processes of autophagy and malignant biological behaviors in GC cells under GD conditions.

## Data availability statement

The datasets presented in this study can be found in online repositories. The names of the repository/repositories and accession number(s) can be found in the article/**Supplementary Material**.

## Author contributions

MZ and CW administrated the whole research. HW and SH performed the experiments. LC and XWL completed the figures. XHL, XQL and XC revised the manuscript and adjusted the layout of figures. The paper was co-written by LF and GQ. All authors contributed to the article and approved the submitted version.
